# Multi-center validation of a machine learning model for early detection of monoclonal immunoglobulin-related disorders using routine laboratory data

**DOI:** 10.3389/fimmu.2026.1856505

**Published:** 2026-06-16

**Authors:** Yujiao Hu, Xiaoyan Hao, Xiaoyan Li, Hailong Tang, Ruixi Liu, Jianrui Yang, Weihua Zhang, Juan Wang, Xinran Liu, Yitong Zhou, Ying Zhang, Jun Zhang, Yanjun Diao, Lei Zhou

**Affiliations:** 1 Department of Clinical Laboratory Medicine, Xijing Hospital, Fourth Military Medical University, Xi’an, China; 2 Department of Hematology, Xijing Hospital, Fourth Military Medical University, Xi’an, China; 3 College of Clinical Medicine, Xi’an Medical University, Xi’an, China; 4 Department of Clinical Laboratory Medicine, 3201 Hospital, Hanzhong, China; 5 Department of Hematology, Shaanxi Provincial People’s Hospital, Xi’an, China; 6 School of Computer Science, Xianyang Normal University, Xianyang, China

**Keywords:** immunofixation electrophoresis (IFE), light gradient boosting machine (LightGBM), machine learning, monoclonal immunoglobulin (MIg), routine laboratory data

## Abstract

**Background:**

Monoclonal immunoglobulin (MIg)-related disorders are clonal plasma cell diseases defined by aberrant MIg overproduction, typically presenting with subtle, non-specific manifestations that lead to underrecognition in routine clinical practice, delayed diagnosis, and subsequent irreversible organ damage and poor outcomes.

**Methods:**

To address this critical gap and facilitate timely MIg diagnosis, we developed and compared eight machine learning models using clinical and routine laboratory data. Cohort 1 (n=5018) included patients undergoing their first serum immunofixation electrophoresis (IFE) test at Xijing Hospital between 2017 and 2024, comprising 2038 MIg-positive and 2980 MIg-negative cases. The top-performing model was subsequently advanced for Cohort 2 (n=2270), consisting of three subsets (Set 1: Xijing Hospital, n=1753; Set 2: 3201 Hospital, n=398; Set 3: Shaanxi Provincial People’s Hospital, n=119), served as a fully independent multi-institutional cohort, and was applied to assess the generalizability of the model.

**Results:**

Feature selection identified ten key variables–urinary cast count, mean corpuscular volume, prothrombin time, activated partial thromboplastin time, albumin/globulin ratio, globulin, total protein, calcium, age, and sex–which were used for final model construction. Among the eight machine learning algorithms, the light gradient boosting machine (LightGBM) model showed superior performance, achieving an AUC of 0.945 (87.0% sensitivity, 89.6% specificity) in the Cohort 2, and achieved 84.6% sensitivity, significantly outperforming the conventional serum protein electrophoresis (75.3% sensitivity) in the subset of cohort 2. In a separate cohort of newly enrolled patients, its 88.2% sensitivity also exceeded that of three non-specialist clinicians.

**Conclusions:**

This LightGBM model, built on readily available clinical and routine laboratory data, may help identify potential MIg-related cases and prioritize patients for further specialized confirmatory testing, which could facilitate earlier recognition and reduce diagnostic delays.

## 1 Introduction

Monoclonal immunoglobulin (MIg)-related disorders are a heterogeneous group of clonal plasma cell disorders defined by aberrant overproduction of MIg (1). Owing to their subtle, non-specific early manifestations, these disorders are frequently overlooked in general clinical practice, leading to substantial diagnostic delay (2). This delay commonly culminates in irreversible organ damage and poor clinical outcomes, underscoring a critical need for accessible, early screening strategies (3, 4).

The current clinical screening pathway for MIg (5, 6) relies on the sequential application of serum protein electrophoresis (SPE) and immunofixation electrophoresis (IFE)—the gold standard for MIg confirmation—supplemented by serum free light-chain (sFLC) analysis. Limitations of the current pathway, including ill-defined screening indications due to highly variable clinical presentation of MIg-related disorders, the invasiveness of bone marrow aspiration, and global disparities in resource access. In real-world practice, however, MIg-related disorders are frequently overlooked in nonspecialist settings, and appropriate screening tests are often not initiated at all (7, 8). Consequently, many patients are not referred for definitive workup until advanced organ damage is apparent. In the contemporary clinical landscape, where effective therapeutic interventions are now available to prevent severe organ impairment, including lytic bone disease, pathological fractures, kidney injury, and life-threatening infection (9, 10). Timely identification of underlying MIg pathology enables optimal utilization of these therapeutic modalities, thereby mitigating the risk of irreversible tissue damage and improving long-term clinical outcomes for affected patients (11, 12).

Machine learning (ML) models can identify subtle patterns and complex relationships in high-dimensional data that conventional assessment often misses, making them promising for early prediction and risk stratification of MIg-related disorders. Wennmann et al. combined deep learning and radiomics to non-invasively predict bone marrow biopsy findings in multiple myeloma (MM) using MRI imaging, achieving an AUC of 0.86 and enabling non-invasive disease staging and risk stratification (13). Cai et al. performed a retrospective study involving 465 multiple myeloma patients and 150 healthy controls, utilizing LASSO to select six parameters from routine blood count and cell population data for model development. The random forest model, which demonstrated the best performance with an AUC of 0.875 in the test set, was proposed as a valuable auxiliary tool for rapid MM screening (14). Yan et al. developed an AI-assisted diagnostic model for MM using routine hematological and biochemical data from 4187 clinical records, which included non−routine markers such as immunoglobulins alongside conventional laboratory parameters (15).

While these works demonstrate the potential of ML, existing studies have several critical limitations that hinder their utility in early detection of MIg-related disorders. Most research focuses on established MM rather than the early stages of MIg-related disorders before severe organ or tissue damage occurs, leaving the key clinical demand for early intervention unmet. In addition, many models rely on specialized data such as radiomic features or non-routine markers including immunoglobulins, which are not widely available in general clinical settings. Furthermore, few studies include multicenter external validation or direct comparisons with routine screening tests and non-specialist clinicians, restricting real-world generalizability and clinical translation. These gaps highlight an urgent need for a robust, widely applicable model for early and accurate identification of MIg-related disorders using routinely available clinical data.

Given these limitations, together with the fact that awareness and recognition of MIg-related disorders are largely limited to specialized hematology and nephrology units, leading to high rates of misdiagnosis and underdiagnosis in other departments, the present study aimed to explore a proof-of-concept approach for the early screening and early warning of MIg-related disorders. Using only widely available routine clinical and laboratory data, we developed, compared, and independently validated a multi-center ML model that enables timely and accessible screening in non-specialized settings, with the goal of supporting earlier recognition and reducing diagnostic delays.

## 2 Methods

### 2.1 Model development cohort

The primary cohort for model development was retrieved from all subjects who underwent serum IFE at Xijing Hospital from July 2017 to December 2024. Concomitantly, routine laboratory data corresponding to the timing of IFE were collected. To ensure that the data accurately reflect the same clinical status of each subject at the time of IFE, the interval between routine laboratory tests and IFE was restricted to ≤7 days. Routine laboratory data included, but were not limited to, complete blood count (CBC), liver function tests (LFTs), renal function tests (RFTs), electrolyte panels, and coagulation function assays.

To avoid potential data leakage, a strict temporal split and independent multi-center validation design was adopted. All model training, feature selection, and data preprocessing were performed solely on the model development cohort (July 2017 to December 2024). External validation cohorts, including the 2025 cases from Xijing Hospital and those from other independent centers, were not involved in any training or feature optimization procedures. All preprocessing rules were fitted on the training cohort only and then directly applied to validation sets without refitting.

### 2.2 Inclusion and exclusion criteria for model development

#### 2.2.1 Inclusion criteria

(1) Subjects with complete clinical and routine laboratory data; (2) Routine laboratory data free of obvious interfering artifacts, such as invalid results caused by specimen hemolysis, lipemia, improper storage, or delayed submission; (3) Data collected in accordance with the principle of one IFE test paired with one set of concurrent routine laboratory data to eliminate cross-timepoint data confounding; (4) IFE results independently interpreted and mutually confirmed by at least two qualified laboratory professionals, with data entered using a double-entry verification method to minimize transcription errors.

#### 2.2.2 Exclusion criteria

(1) Not-first serum IFE records from subjects who underwent multiple IFE tests during the study period; (2) Subjects with only IFE results without completion of the specified concurrent routine laboratory tests; (3) Subjects with ambiguous IFE results or inconsistent interpretations between the two reviewing professionals that could not be resolved through joint re-evaluation.

### 2.3 Statistical analysis

Statistical analysis was performed using SPSS 23.0 (IBM Corp., NY, USA). The sample distribution was determined using the Kolmogorov-Smirnov normality test. Non-normally distributed continuous data were expressed as median (M) and interquartile range (IQR) in the format of “M[IQR]”, and intergroup comparisons were conducted using the Mann-Whitney U test. Categorical data were presented as number of cases and percentage in the format of “n (%)”, and comparisons of percentages between groups were performed using the chi-square test. *P* < 0.05 was considered indicative of statistical significance. Machine learning models were developed using Python version 3.7 (Python Software Foundation, DE, USA). Data visualizations were performed using R version 4.2.3 (R Foundation for Statistical Computing, Vienna, Austria).

### 2.4 Data preparation

#### 2.4.1 Missing value handling

First, laboratory test items with a missing rate exceeding 30% across all included subjects were excluded. For remaining items with missing values, imputation was performed using the random forest algorithm. This approach constructs an ensemble of decision trees to exploit inherent inter-feature correlations, enabling predictive imputation of missing values while maximizing data retention (16).

#### 2.4.2 Multicollinearity analysis

To mitigate the impact of multicollinearity on subsequent analyses, all test items underwent collinearity assessment via variance inflation factor (VIF) computation (17). Items with VIF > 5, indicating substantial collinearity, were excluded to mitigate multicollinearity. Specifically, for sets of variables with high mutual collinearity, redundant items within each collinear group were selectively removed as appropriate, while the remaining non-redundant variables were retained for downstream modeling.

### 2.5 Feature selection

Five algorithms, least absolute shrinkage and selection operator (LASSO), relevant feature selection algorithm F (ReliefF), minimum-redundancy maximum-relevance (mRMR), Boruta, and recursive feature elimination (RFE) were applied to perform feature selection on the Cohort 1 (18, 19).

### 2.6 Development of the machine learning model

Cohort 1 was utilized for feature selection, alongside the construction, hyperparameter tuning, and testing of the developed models. Leveraging routine clinical data encompassing conventional laboratory blood test parameters, we developed and systematically compared eight ML models, including extreme gradient boosting (XGBoost), the light gradient boosting machine (LightGBM), gradient boosting decision tree (GBDT), random forest (RF), logistic regression (LR), gaussian naive bayes (GNB), multilayer perceptron (MLP), and support vector machine (SVM) for predicting the risk of MIg positivity.

For model development (20), the cohort (N = 5018) was randomly split into a training set (90%, n = 4516) and an independent test set (10%, n = 502). Ten-fold cross-validation was performed on the training set for hyperparameter tuning. The LightGBM model was trained with the following parameters: boosting_type=‘gbdt’, learning_rate=0.09, n_estimators=100, num_leaves=29, max_depth=-1, min_child_samples=20, colsample_bytree=1.0, subsample=1.0, reg_alpha=0.0, and reg_lambda=0.0. Given the mild class imbalance (negative/positive ≈ 1.46:1), no resampling was performed.

Ultimately, the model exhibiting the optimal performance in both the internal validation sets and the test set was selected for subsequent analysis.

### 2.7 Multi-institutional independent external validation

For an unbiased and rigorous independent external validation, Cohort 2 was composed of datasets from three distinct hospitals, with no overlap with the cohort used for model development. This multi-institutional validation design enabled a comprehensive assessment of the stability and generalizability of our diagnostic model. Specifically, set 1: 1753 cases collected from Xijing Hospital between January 2025 and August 2025; set 2: 398 cases derived from 3201 Hospital from 2024 to 2025; set 3: 119 cases obtained from Shaanxi Provincial People’s Hospital from 2024 to 2025.

### 2.8 Comparison of the discriminative capacity between the ML and SPE

Among subjects in the external validation Cohort 2, a subset of 804 cases had concurrent SPE results, a conventional screening method for MIg, in addition to their IFE results and routine laboratory data. A head-to-head comparison was performed between the ML model and SPE in this subset to assess their diagnostic performance for detecting MIg positivity, with IFE results adopted as the reference standard.

### 2.9 Comparison of diagnostic performance between clinicians and the LightGBM model in newly recruited cases

To further assess the temporal stability and clinical applicability of the pre-trained LightGBM model, an independent time-extended external validation cohort was recruited. This cohort included 627 consecutive patients who underwent IFE and routine laboratory testing at Xijing Hospital between September and December 2025, after the cutoff date of the original training and validation cohorts.

For the newly recruited cases, the prediction of IFE positivity was independently performed by three clinicians without specialized training in hematology or nephrology. Their assessments were based on comprehensive basic clinical information and complete sets of routine laboratory data of each patient. Notably, all specialized diagnostic tests that could directly or indirectly indicate the presence of MIg—including SPE, sFLC analysis, and immunoglobulin quantitative detection—were excluded from the materials provided to the clinicians to avoid explicit hints of MIg positivity.

Concurrently, predictive probabilities for each patient were generated by the LightGBM model using the optimal threshold derived from the original validation cohort. To eliminate subjective bias, the actual IFE results (serving as the gold standard for MIg confirmation) and the predictive outputs of the model were blinded to all participating clinicians. The diagnostic performance of the clinicians and the LightGBM model were subsequently compared, with sensitivity, specificity, area under the curve (AUC), and Kappa coefficient as the primary evaluation metrics.

**Figure 1:**
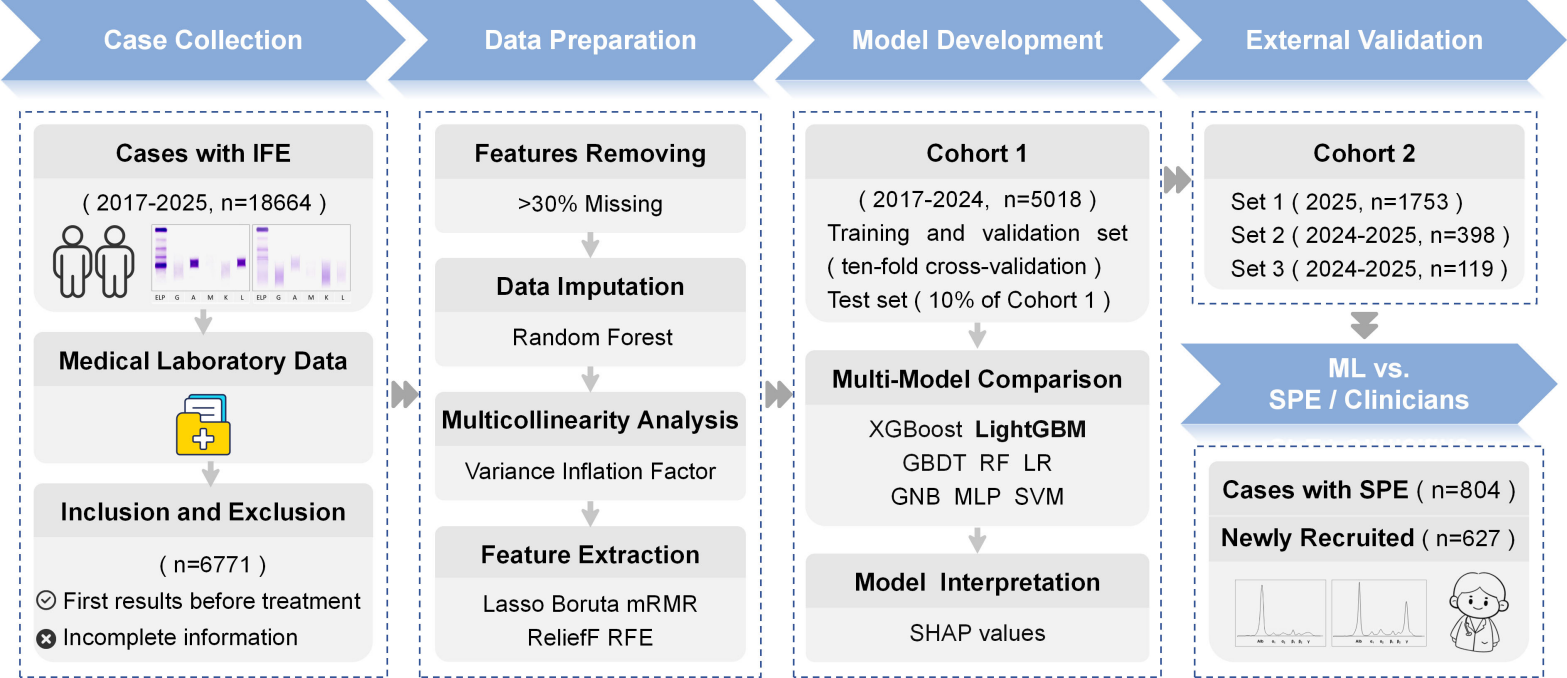
Schematic overview of the study workflow for MIg prescreening using machine learning models.

The methodology applied in this study is illustrated in the workflow shown in **Figure 1**.

### 2.10 Development of the online prediction platform

To facilitate the convenient auxiliary application of the model in clinical research and practice settings, we developed a user-friendly web-based prediction platform available at https://online.predmodel.com/migdx.

The platform adopts a front-end and back-end separated architecture. The interface is built with HTML, and the trained LightGBM model is serialized and stored in Python pickle format. The platform integrates standardized input validation for data type, reasonable value range and logical consistency, and does not perform automatic imputation for missing required inputs.

## 3 Results

### 3.1 Baseline characteristics

**Figure 2:**
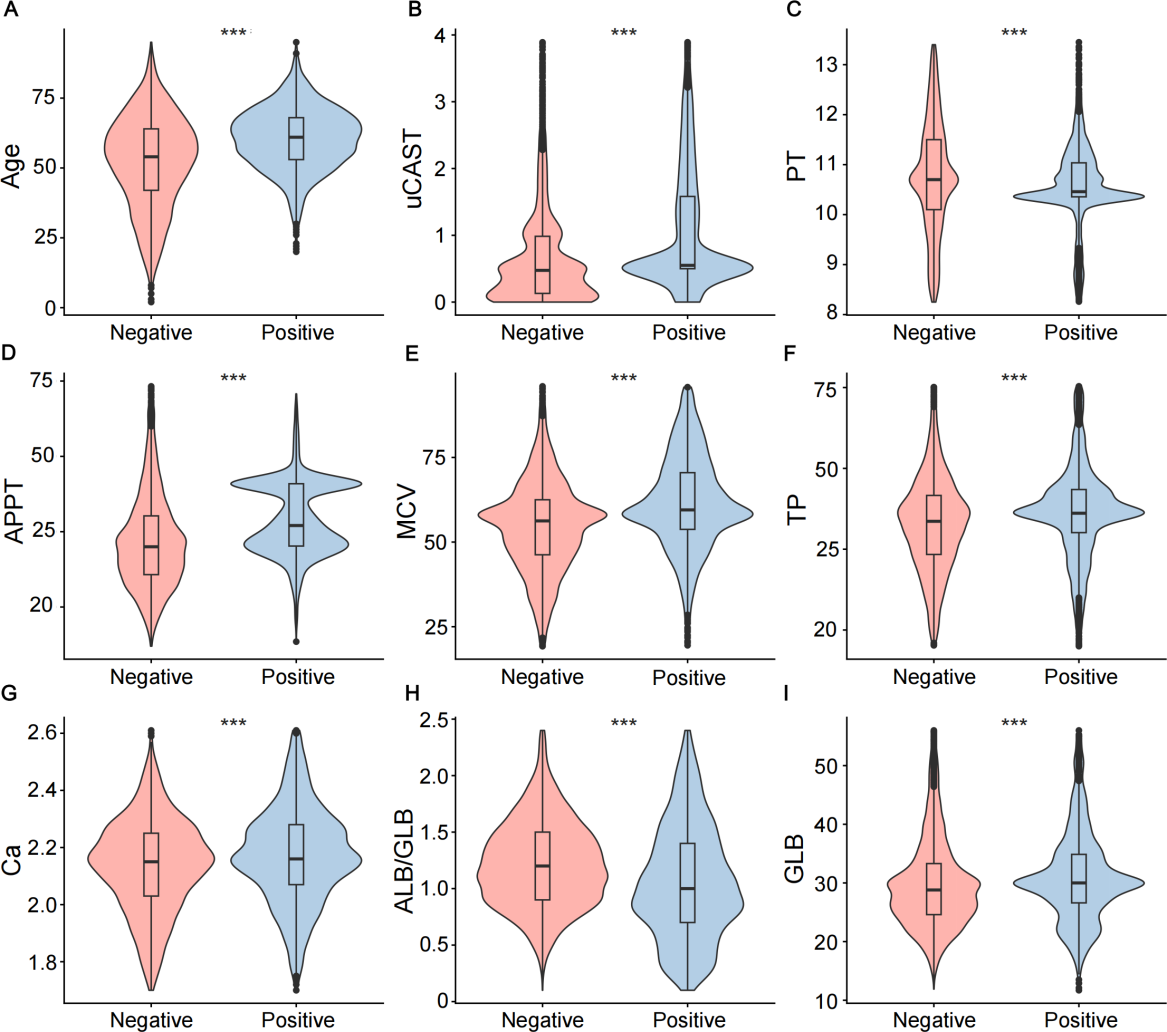
Violin plots comparing distributions between IFE positive and negative groups. (uCAST, urine casts; PT, prothrombin time; APTT, activated partial thromboplastin time; MCV, mean corpuscular volume; TP, total protein; Ca, calcium; ALB/GLB, albumin/globulin ratio; GLB, globulin). ***p < 0.001.

From 18664 IFE results and corresponding routine laboratory data collected over a 9-year period at Xijing hospital, a total of 6771 eligible subjects were enrolled in this study, including 2708 IFE-positive and 4063 IFE-negative cases. A total of 5018 subjects, including 2038 IFE-positive and 2980 IFE-negative, were enrolled from 2017 to 2024 as the model development Cohort 1. The baseline characteristics of Cohort 1 are summarized in **Supplementary Table 1**, **Figure 2**.

### 3.2 Feature selection

**Figure 3:**
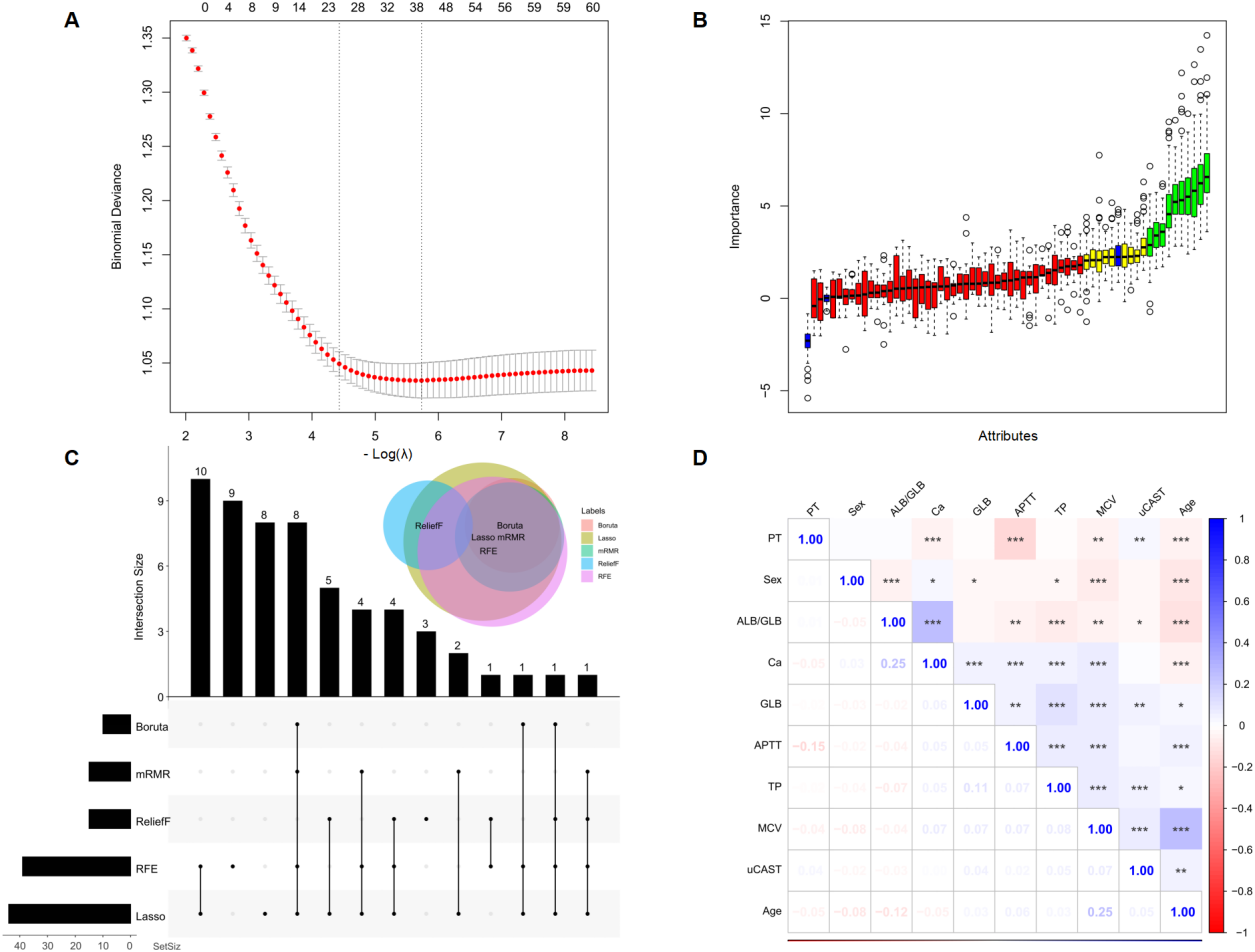
Feature selection analyses for identifying key variables. **(A)** Binomial deviance curve for gradient boosting model during feature optimization. **(B)** Variable importance ranking of candidate features based on their contribution to gradient boosting model performance. **(C)** Venn Diagram of overlapping features identified by multiple analytical strategies. **(D)** Heatmap of Spearman correlation coefficients between candidate features. * = p < 0.05, ** = p < 0.01, *** = p < 0.001.

To assess the most discriminative features for identifying IFE-positivity, a comprehensive feature selection process was conducted (**Figure 3**). Subsequent screening yielded 44 features selected by LASSO, 10 features selected by Boruta, 15 features selected by mRMR, 15 features selected by ReliefF, and 39 features selected by RFE (**Figure 3A**; **Supplementary Table 2**). Ultimately, a synthesis of results from multiple feature selection methods, including Boruta, mRMR, ReliefF, RFE, and LASSO, identified a consensus set of key features, among which sex, age, urinary cast (uCAST), mean corpuscular volume (MCV), prothrombin time (PT), activated partial thromboplastin time (APTT), albumin/globulin ratio (ALB/GLB), globulin (GLB), total protein (TP) and calcium (Ca) were consistently prioritized by at least four methods, underscoring their robust relevance and potential as core predictors for MIg diagnosis (**Figure 3**). The correlation heatmap revealed minimal multicollinearity among the candidate features as indicated by correlation coefficients mostly below 0.3. This ensured the robustness of subsequent feature selection (**Figure 3**).

### 3.3 Model development

#### 3.3.1 Multi-model performance comparison

**Figure 4:**
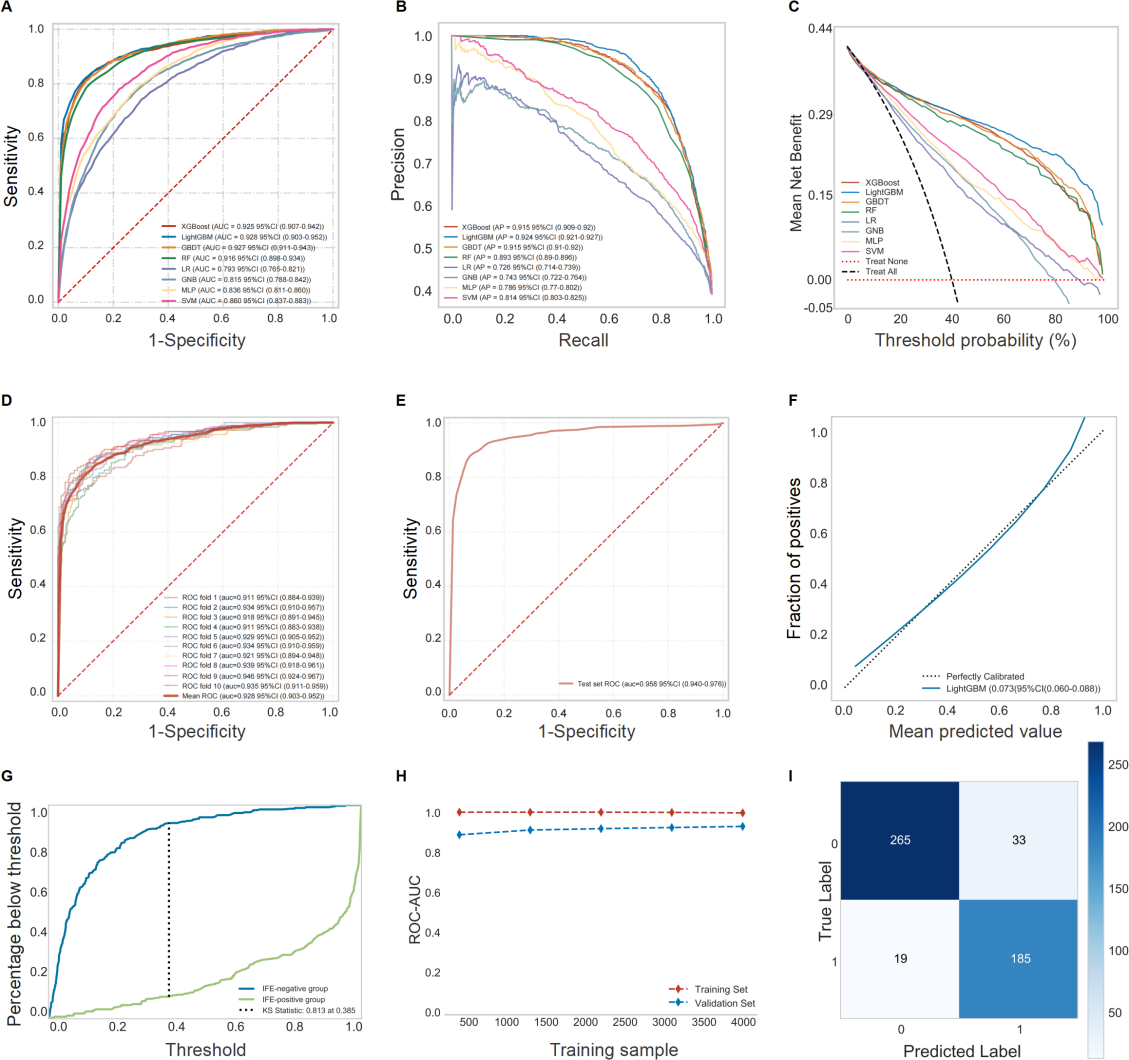
Model performance of ML algorithms. **(A)** ROC curves of internal validation set for multiple ML models. **(B)** Precision-recall curves of internal validation set for multiple ML models. **(C)** Mean net benefit curves of internal validation set for multiple ML models across varying threshold probabilities, with the dashed line representing the net benefit of a strategy of treating all patients. **(D)** Ten-fold cross-validation ROC curve of the LightGBM model in the internal validation set. **(E)** ROC curve of test set for the LightGBM model. **(F)** Calibration curve of the LightGBM model. **(G)** Threshold analysis curve of the LightGBM model, illustrating the percentage of cases below threshold across different threshold values. **(H)** ROC-AUC curve stability analysis of the LightGBM model. **(I)** Confusion matrix of test set for the LightGBM model. The color scale indicates the number of cases corresponding to each combination of predicted and true labels.

To evaluate the diagnostic efficacy of ML models for MIg using routine laboratory data, multiple algorithms were compared, with performance primarily assessed on the internal validation set. ROC analysis revealed LightGBM achieved the highest AUC among all tested models (**Figure 4**). Consistently, precision-recall (PR) curve analysis confirmed LightGBM yielded the highest average precision (AP) (**Figure 4**). Decision curve analysis further supported LightGBM’s clinical utility, as it provided the highest net benefit across a broad range of threshold probabilities—outperforming other models and hypothetical “Treat All” or “Treat None” strategies (**Figure 4**). Overall, these results indicate LightGBM exhibits superior discriminative capacity and clinical applicability for MIg diagnosis based on routine laboratory data, compared to other commonly used ML algorithms.

#### 3.3.2 LightGBM model

The LightGBM model showed promising performance in cross-validation and test phases, and preliminary evidence of stability was obtained from subsequent iterative analyses. The cross-validation ROC curves of the LightGBM model exhibited high consistency across iterations, indicating robust and stable discriminative performance of the model (**Figure 4**). In the internal validation set, it achieved a mean AUC of 0.928 with a 95% confidence interval (CI) of 0.903-0.952, confirming reliable discriminative ability for MIg. When evaluated on the test set, the model further demonstrated excellent preliminary generalizability, with an AUC of 0.958 with a 95% CI of 0.940-0.976 (**Figure 4**). The calibration plot showed good alignment between predicted probabilities and actual clinical outcomes, supporting the reliability of the model’s predictions (**Figure 4**). The KS statistic plot with a KS value of 0.813 at the optimal threshold, highlighted the model’s strong separation of the IFE-positive group (**Figure 4**). Learning curves illustrated that with the expansion of training sample size, the ROC-AUC of both the training and internal validation sets maintained consistent stability, and the two curves exhibited tight convergence (**Figure 4**). This observation not only verified the model’s stable predictive performance across varying sample sizes but also confirmed the absence of substantial overfitting. The confusion matrix detailed the model’s predictive accuracy on the test set (**Figure 4**). Collectively, these results confirm that the LightGBM model maintains high diagnostic accuracy, reliability, and potential clinical applicability for MIg diagnosis using routine laboratory data, laying a foundation for subsequent independent external validation (**Table 1**).

**Table 1:** The LightGBM performance across different datasets.

Diagnostic indicators	Model development cohort 1 (n=5018)			Independent external validation cohort 2 (n=2270)			
	Training set (n=4065)	Validation set (n=451)	Test set (n=502)	Cohort 2 (total n=2270)	Set 1 (n=1753)	Set 2 (n=398)	Set 3 (n=119)
Sensitivity (95% CI)	0.975 (0.972-0.978)	0.832 (0.820-0.844)	0.907 (0.900-0.914)	0.870	0.867	0.833	0.880
Specificity (95% CI)	0.973 (0.970-0.975)	0.877 (0.864-0.890)	0.889 (0.882-0.896)	0.896	0.873	0.984	0.942
Accuracy (95% CI)	0.974 (0.973-0.975)	0.859 (0.848-0.870)	0.896 (0.889-0.903)	0.888	0.871	0.975	0.916
PPV (95% CI)	0.961 (0.957-0.964)	0.823 (0.807-0.840)	0.849 (0.842-0.856)	0.804	0.809	0.769	0.917
NPV (95% CI)	0.983 (0.981-0.984)	0.884 (0.876-0.892)	0.933 (0.926-0.940)	0.934	0.914	0.989	0.915
AUC (95% CI)	0.997 (0.996-0.997)	0.928 (0.903-0.952)	0.958 (0.940-0.976)	0.945 (0.935-0.955)	0.938 (0.927-0.950)	0.977 (0.951-1.000)	0.946 (0.897-0.994)
F1-Score (95%CI)	0.968 (0.966-0.969)	0.827 (0.815-0.840)	0.877 (0.870-0.884)	0.835	0.837	0.800	0.898
Kappa (95%CI)	0.945 (0.943-0.948)	0.708 (0.687-0.730)	0.788 (0.780-0.796)	0.750	0.731	0.787	0.827

For the training set, validation set, and test set in Cohort 1, 95% confidence intervals (CIs) are provided alongside the metrics.

For the independent external validation set (Cohort 2), metrics are presented as point estimates, except for AUC, where the 95% CI was visualized from the ROC curve.

### 3.4 Model interpretation

**Figure 5:**
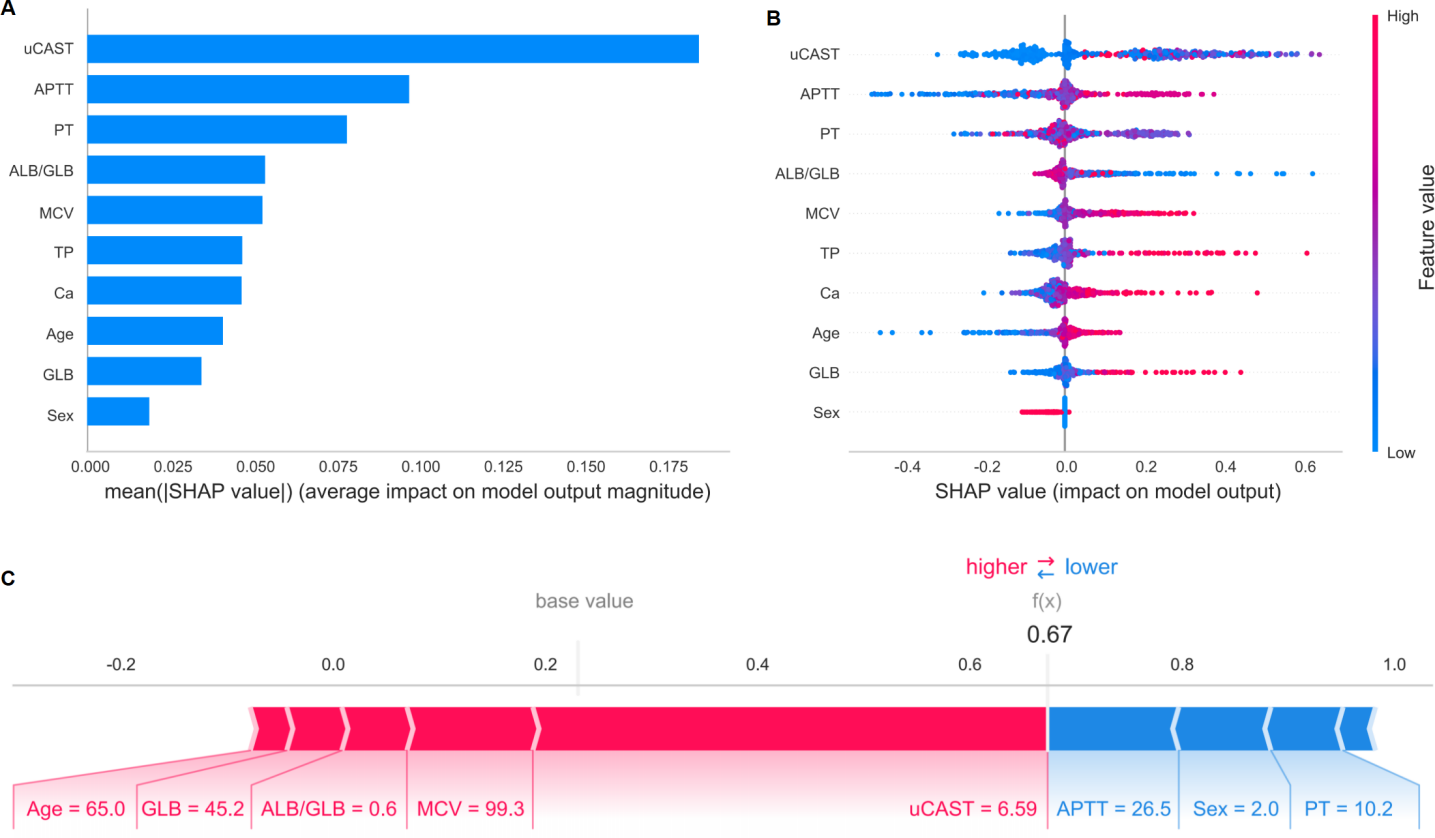
Model interpretability analysis using SHAP values for the ML model. **(A)** Feature importance ranking based on mean SHAP values. **(B)** SHAP summary plot, showing the relationship between feature values and their corresponding SHAP values. **(C)** SHAP force plot for a representative sample.

To elucidate the interpretability of the LightGBM model for MIg diagnosis, SHapley Additive exPlanations (SHAP) analysis was performed to quantify the contribution of each feature to model predictions. The mean SHAP value plot revealed uCAST had the highest average impact on the model output, followed by activated partial thromboplastin time APTT, prothrombin time PT, and ALB/GLB, indicating these features are the most influential predictors in the model (**Figure 5**). The SHAP summary plot further illustrated the direction and magnitude of feature impacts. For instance, higher uCAST levels were associated with a positive impact on the model’s prediction of MIg, while higher APTT values also contributed positively, whereas a higher ALB/GLB ratio had a negative impact (**Figure 5**). The SHAP force plot provided a case-specific interpretation, decomposing the model’s prediction f(x) = 0.67 into contributions from individual features, highlighting that uCAST (6.59/µL) and APTT (26.5 s) were the primary drivers of this prediction, while other features like Age (65 years), PT (10.2 s), MCV (99.3 fL) and ALB/GLB (0.6) also played roles in shaping the final output (**Figure 5**). These results not only confirm the model’s interpretability but also identify key laboratory indicators, that clinicians can prioritize when screening for MIg, bridging the gap between ML performance and clinical decision-making.

### 3.5 Generalizability of the model in multi-center independent external validation

**Figure 6:**
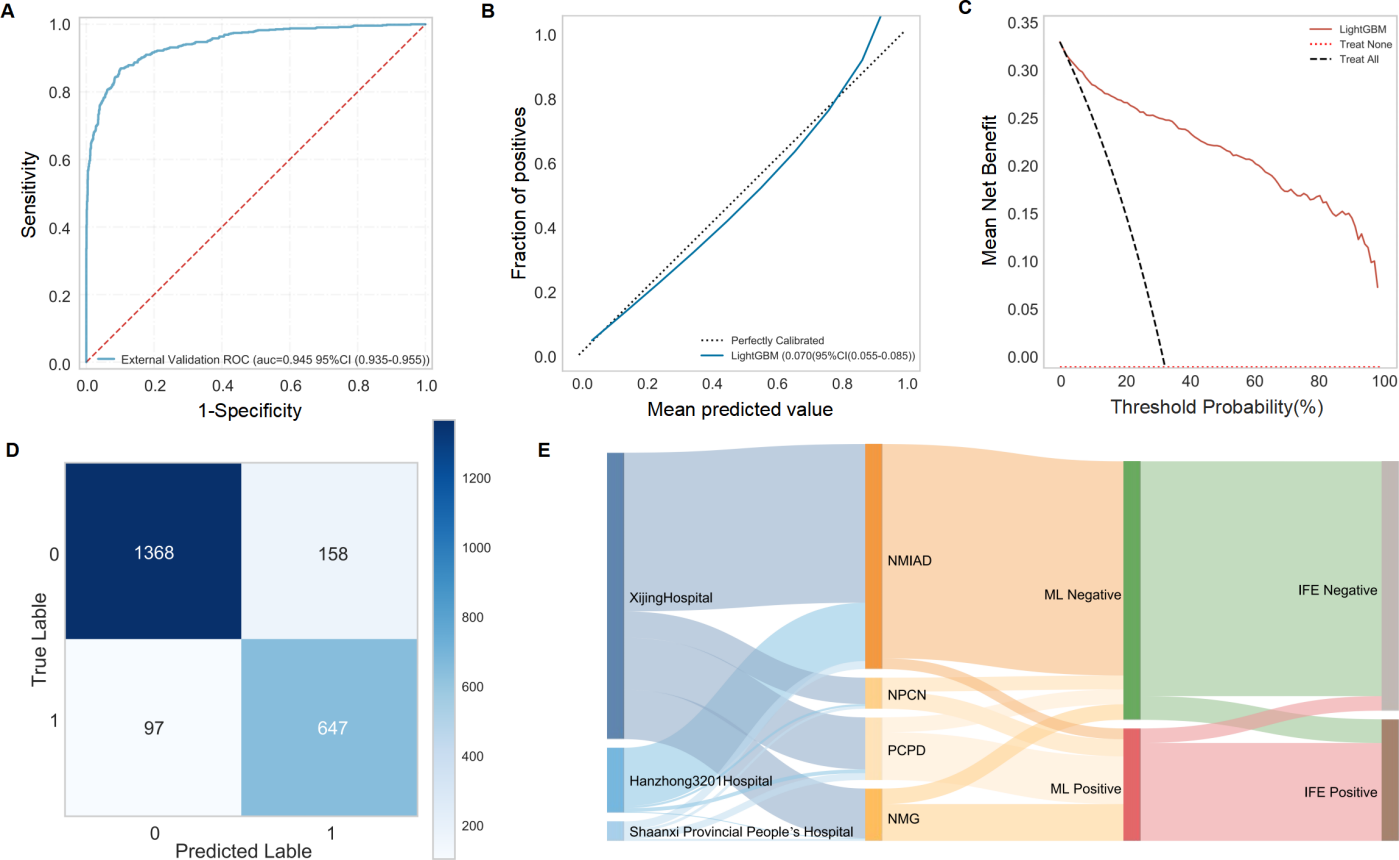
Performance evaluation of the ML model in independent external validation. **(A)** ROC curve of the model in independent external validation. **(B)** Calibration curve of the LightGBM model in independent external validation. **(C)** Mean net benefit curve of the model across varying threshold probabilities. **(D)** Confusion matrix of the model in independent external validation. **(E)** Visualization of 2270-sample flow across four sequential levels: data sources (3 hospitals), disease groups (NMIAD, non-monoclonal-expected immunoglobulin abnormality disorders group; NPCN, non-plasma cell-derived neoplasms group; PCPD, plasma cell proliferative disorders group; NMG, non-neoplastic monoclonal gammopathy group), IFE results (negative/positive), and model predictions (negative/positive). Link width reflects the sample volume for each flow.

The LightGBM model demonstrated robust and generalizable diagnostic performance in the independent multi-center external validation cohort (Cohort 2, n=2270), extending its utility beyond the development dataset. First, the model retained excellent discriminative capacity, with an AUC of 0.945 (95% CI: 0.935 -0.955), confirming its ability to accurately identify patients with MIg positivity (**Figure 6**). Calibration analysis further validated the model’s reliability, showing strong alignment between predicted probabilities and actual outcomes, with a non-significant Hosmer-Lemeshow test (*P* > 0.05) (**Figure 6**). Decision curve analysis reinforced its clinical value: across a broad range of clinically relevant threshold probabilities, the model delivered higher net benefit than both “Treat All” and “Treat None” strategies, supporting its potential to guide clinical decision-making (**Figure 6**). At the optimal cutoff, the model achieved 87.0% sensitivity, 89.6% specificity, 88.8% accuracy, 80.4% positive predictive value, and 93.4% negative predictive value (**Table 1**). Critically, subgroup analyses across three independent centers revealed consistent performance (AUC range: 0.938-0.977), with minimal variation in sensitivity and specificity across heterogeneous patient populations. Sankey diagram visualization further corroborated this consistency, demonstrating uniform model performance across diverse data sources, disease subtypes, and IFE result categories (**Figure 6**).

Taken together, these findings confirm that the LightGBM model maintains high diagnostic accuracy and stability in multi-center external validation, supporting its potential for broad clinical implementation across diverse healthcare settings.

### 3.6 ML model outperforms SPE in discriminative performance

Among the 1753 subjects in the independent external validation cohort, 804 had concurrent SPE results, a well-established first-line screening tool for MIg in clinical practice. For the detection of MIg with IFE as the reference standard, the ML model demonstrated a significantly higher sensitivity of 84.6% compared to 75.3% for SPE, while maintaining a favorable specificity of 90.9% against SPE’s 95.6% (**Table 2**). Although SPE exhibited marginally higher specificity, the ML model’s robust balance of SEN and SPE, coupled with its reliance on readily available routine laboratory data, renders it a valuable complementary or alternative screening tool, particularly in settings where MIg is prone to being overlooked or missed.

**Table 2:** Diagnostic performance comparison between the ML model and SPE.

Method	Sensitivity (95% CI)	Specificity (95% CI)	Accuracy (95% CI)	PPV (95% CI)	NPV (95% CI)	F1-score (95% CI)	Kappa (95% CI)
LightGBM Model	0.846 (0.806-0.879)	0.909 (0.878-0.932)	0.879 (0.855-0.900)	0.891 (0.855-0.919)	0.870 (0.836-0.898)	0.868 (0.842-0.894)	0.757 (0.710-0.798)
SPE	0.753 (0.707-0.794)	0.956 (0.932-0.971)	0.861 (0.835-0.883)	0.937 (0.904-0.959)	0.814 (0.778-0.846)	0.835 (0.804-0.864)	0.717 (0.669-0.765)

It enhanced the identification of patients with occult or early-stage MIg positivity, individuals who might otherwise be overlooked in routine clinical practice. This advantage is clinically meaningful, as timely detection of MIg is pivotal for initiating early interventions, optimizing patient management, and improving long-term outcomes.

### 3.7 LightGBM model surpasses clinicians in new-case diagnostic efficacy

The time-extended independent validation cohort used in this study, although not a strictly prospective design, we effectively demonstrated that the model’s performance was not limited to historical data but could be generalized to newly recruited cases.

The LightGBM model outperformed clinicians of different experience levels in detecting MIg-positive cases, with notable advantages in sensitivity and the ability to balance false positives and false negatives (**Table 3**). This supplementary validation confirms that the LightGBM model is not merely a data-fitted tool but a practical clinical auxiliary that complements rather than replaces clinicians’ expertise. The model’s superior sensitivity, especially in identifying occult and early-stage cases, directly assists in addressing the key limitation of experience-dependent clinical judgment, particularly for non-specialized physicians or those with limited familiarity with MIg-related disorders. Given the multi-departmental distribution of MIg-related cases (**Supplementary Table 3**), the model’s consistent performance across different clinical settings further supports its potential as a preliminary screening aid to flag high-risk individuals for specialized evaluation. This model-clinician synergy not only reduces the risk of diagnostic delays but also optimizes the allocation of medical resources, aligning with the goal of precise and efficient clinical practice.

**Table 3:** Diagnostic performance comparison between the ML model and clinicians.

Predictor	Sensitivity (95% CI)	Specificity (95% CI)	Accuracy (95% CI)	PPV (95% CI)	NPV (95% CI)	F1-score (95% CI)	Kappa (95% CI)
LightGBM Model	0.882 (0.835-0.920)	0.875 (0.842-0.903)	0.877 (0.850-0.901)	0.737 (0.684-0.785)	0.949 (0.926-0.967)	0.803 (0.760-0.841)	0.736 (0.678-0.794)
Clinician 1	0.815 (0.756-0.865)	0.748 (0.708-0.785)	0.767 (0.732-0.799)	0.562 (0.505-0.618)	0.911 (0.882-0.935)	0.665 (0.613-0.713)	0.528 (0.453-0.603)
Clinician 2	0.742 (0.676-0.800)	0.759 (0.720-0.796)	0.754 (0.719-0.787)	0.550 (0.493-0.606)	0.881 (0.848-0.908)	0.632 (0.579-0.682)	0.412 (0.335-0.489)
Clinician 3	0.697 (0.628-0.759)	0.731 (0.690-0.768)	0.721 (0.685-0.755)	0.506 (0.450-0.562)	0.859 (0.823-0.889)	0.586 (0.532-0.638)	0.386 (0.308-0.464)

## 4 Discussion

MIg-related disorders pose substantial challenges to routine clinical diagnosis, with a critical yet underrecognized barrier rooted in limited awareness among non-specialist clinicians and patients, rather than the lack of available screening tools. Although conventional detection methods such as SPE and IFE are well established, non-specialist practitioners may often overlook subtle clinical clues suggestive of MIg-related diseases, while patients frequently disregard non-specific early symptoms. Such diagnostic inertia may lead to delayed diagnosis and treatment, accelerate disease progression, and ultimately impair long-term prognostic outcomes.

Our findings further underscore the pervasiveness of this diagnostic burden: as presented in **Supplementary Table 3**, IFE-positive cases were widely distributed across multiple clinical departments, led by Hematology, Nephrology, and Emergency Medicine. Considerable positive cases also initially presented to many other non-specialty departments, including Immunology, Cardiology, Neurology, Gastroenterology, Orthopedics, Oncology, Dermatology, Trauma, and other departments. This extensive interdepartmental dispersion reflects the heterogeneous and non-specific manifestations of MIg-related disorders, which could easily mimic common conditions such as renal insufficiency, arthralgia, or anemia, thereby raising the risk of misdiagnosis or delayed specialized care. Collectively, these data highlight an urgent unmet clinical need for a simple, widely applicable tool to enable early identification of MIg-related disorders in non-specialist settings.

To directly address this potential gap, the present study is, to the best of our knowledge, the first to develop a ML diagnostic model for MIg-related disorders using two categories of routinely accessible, low-cost data: basic demographic information (age and sex) and common laboratory indicators (uCAST, MCV, PT, APTT, ALB/GLB, GLB, TP, Ca).

Notably, all these variables are universally collected in general hospital settings, eliminating the need for additional specialized testing or invasive procedures. It should be acknowledged that all participants enrolled in the current study were ultimately referred for IFE testing at a tertiary medical center, and the study cohort lacked control populations from unselected general outpatient, community, and primary care settings. Further enlarging the sample size and establishing rigorous control groups from broader real-world populations will be necessary in future work to fully validate the generalizability and screening performance of the model.

By leveraging data that are already part of routine clinical workflows, our model may help circumvent some of the key barriers to early MIg detection, namely, the reliance on specialist expertise and advanced diagnostic resources that are not universally available. This design feature may thus offer a practical strategy to potentially mitigate the clinical burden of undiagnosed or late-diagnosed MIg-related disorders. To contextualize the biological relevance of our model’s features, we drew upon established literature to interpret their associations with MIg-related disorders.

The model identified uCAST count as the most influential predictor, consistent with the well-documented nephrotoxic effects of MIgs. Excess sFLCs are a key component of MIgs and interact with uromodulin in the distal nephron to form obstructive casts. These casts induce tubular damage and interstitial fibrosis, pathological changes that represent the hallmark of MIg-associated cast nephropathy (21). It is important to acknowledge that uCAST was quantified via routine automated urine sediment analysis, a primary clinical screening tool with recognized limitations, including moderate concordance with manual microscopy for pathological casts and potential confounding by non-specific debris or pre-analytical variables (22). However, the model’s robust performance despite this indicator’s inherent variability highlights the value of integrating uCAST into a multi-feature ML framework, which leverages complementary data to enhance specificity for MIg-related renal injury beyond single-marker screening.

Coagulation parameters, particularly APTT and PT, emerged as important contributors to model prediction. Mounting evidence indicates that MIgs disrupt hemostasis through diverse mechanisms, resulting in heterogeneous coagulation dysfunction. MIgs may directly bind and inhibit coagulation factors, impair fibrin polymerization, or induce acquired deficiencies of key factors such as factor X, thereby prolonging clotting times (23). Concurrently, MIgs can promote a hypercoagulable state by activating platelets, injuring vascular endothelium, increasing tissue factor expression, or dysregulating natural anticoagulant pathways including the protein C/protein S system (24). Paradoxically, MIgs may also precipitate bleeding diatheses by suppressing platelet aggregation, accelerating factor consumption, or interfering with fibrin cross−linking and clot stabilization (25). Collectively, these dual prothrombotic and hemorrhagic effects translate into variable alterations in PT and APTT—either prolongation or, less commonly, shortening—reflecting the complex and heterogeneous impact of MIgs on the coagulation−fibrinolysis network.

The model also integrated several serum protein indices: GLB, TP and ALB/GLB. These markers directly reflect the underlying plasma cell dyscrasia, where the overproduction of MIgs typically leads to elevated total protein and globulin levels, and a consequent reduction in the ALB/GLB ratio (26, 27). MCV emerged as another key feature. Dysregulated erythropoiesis, often reflected by MCV abnormalities, is common in MIg-related disorders due to bone marrow infiltration, nutrient deficiencies, or the inhibitory effects of cytokines (28–30). Finally, Ca levels were incorporated into the model. Hypercalcemia is a classic paraneoplastic syndrome in MIg-related disorders, primarily driven by bone resorption induced by osteolytic lesions or humoral factors such as parathyroid hormone-related protein (29, 31). All the model leverages routine laboratory markers that are not only easily accessible but also biologically plausible, each reflecting distinct pathogenic pathways in MIg-related disorders.

An interesting and unanticipated observation emerged during the model’s feature selection: despite our proactive measures via VIF screening to mitigate multicollinearity, three indices TP, GLB, and ALB/GLB, that are intuitively expected to be collinear were all retained in the final model. This finding can be primarily attributed to two key factors. First, the feature selection methods employed in our study, inherently excels at resolving multicollinearity by applying penalty terms to compress redundant variable coefficients, thereby retaining only those with independent predictive value and enhancing model interpretability (32, 33). Second, in the context of MIg-related disorders, the elevation of MIgs induces non-linear and heterogeneous variations in serum GLB levels. Specifically, the clonal expansion of plasma cells in MIg-related disorders leads to a disproportionate and variable increase in GLB, which deviates from the proportional changes of ALB and TP in physiological states. Additionally, ALB/GLB, as a derived ratio variable, exhibits an asynchronous distribution and captures unique information regarding the proportional imbalance, rather than merely reflecting redundant information of TP, ALB, or GLB. Collectively, these pathological and statistical characteristics enable TP, GLB, and ALB/GLB to coexist in the predictive model without causing severe multicollinearity.

Despite the promising findings of this study, several limitations should be acknowledged. First, our model was developed using data from a single institution, and although multi-center external validation was performed, this was based on only three independent centers with unbalanced cohort sizes. Consequently, the generalizability of our model may still be constrained, as it may not fully represent the heterogeneity of clinical practices and laboratory standards across a broader range of healthcare settings. Our work should therefore be regarded as a proof-of-concept framework rather than a universally applicable tool. Second, we compared the model with non-specialist clinicians but did not assess its performance against the diagnostic pathway used by specialist clinicians. Future studies should include such comparisons to better delineate the model’s incremental value in routine practice. Third, the model’s performance in certain patient subgroups remains unexplored. Some disease subgroups had relatively small and imbalanced sample sizes, which would lead to unstable and unreliable performance estimates if analyzed separately. In addition, routine IFE only detects IgG, IgA, and IgM subtypes but not IgD or IgE (34), so patients with these rare MIg subtypes cannot be identified by the current model. Fourth, the online platform is currently for academic research and demonstration only. It lacks formal prospective clinical validation and regulatory approval, and is not intended for independent clinical decision-making. Further validation is required prior to clinical application. Finally, this study evaluated diagnostic accuracy only. Whether earlier detection facilitated by the model translates into improved treatment initiation, follow-up adherence, or long-term outcomes remains to be established.

## 5 Conclusion

In conclusion, this study developed a LightGBM-based diagnostic model, which featured data-driven feature selection and interpretable results. The model exhibited stable performance in multi-center independent external validation cohort, achieving an AUC of 0.945, along with a sensitivity of 0.870, specificity of 0.896, accuracy of 0.888, demonstrating reliable diagnostic capability. Although further validation in a broader population is still needed to clarify the practical application value of the model in primary care and general practice settings, as a proof-of-concept tool, it is expected to assist in screening and early warning of potential MIg cases in non-specialized settings using routine clinical and laboratory data, supporting early clinical identification, facilitating timely diagnostic communication, and thereby shortening the duration of diagnostic delays.

## Data Availability

The original contributions presented in the study are included in the article/**Supplementary Material**. Further inquiries can be directed to the corresponding authors.
